# Activity of volatiles induced by microbes and natural plants stifled the growth of *Pythium aphanidermatum* - the damping off in Tomato

**DOI:** 10.1186/s12870-023-04351-3

**Published:** 2023-08-10

**Authors:** Praveen Thangaraj, Krishnamoorthy Akkanna Subbiah, Nakkeeran Sevugapperumal, Sivakumar Uthandi, Amirtham Damodarasamy, Haripriya Shanmugam

**Affiliations:** 1 Department of Plant Pathology, Tamil Nadu Agricultural University, Coimbatore, India; 2 Department of Agricultural Microbiology, Tamil Nadu Agricultural University, Coimbatore, India; 3 Department of Biochemistry and Molecular Biology, Tamil Nadu Agricultural University, Coimbatore, 641 003 India; 4 Department of Nanoscience and Technology, Tamil Nadu Agricultural University, Coimbatore, 641 003 India

**Keywords:** *M. spicata*, *T. asperellum*, Volatilomes, *P. aphanidermatum*, Plant pathogen management

## Abstract

**Background:**

Volatilomes from natural plants and microbes imparts diverse antifungal properties to suppress the growth of plant pathogens and therefore can be a suitable alternative of chemical fungicides. The present experiment was to study effect of volatiles produced by natural plants and microbes on the fungal growth of *Pythium aphanidermatum*, which is a tomato seedling pathogen.

**Results:**

Isolate of *P. aphanidermatum*, causing damping off in tomato were isolated and incubated at 25 ± 2 °C. The isolate was tested for the anti-oomycetes activities of volatiles in vitro*.* The volatiles produced by the leaves of *Mentha spicata* and *Cymbopogon citratus* showed the maximum inhibitory effect of 45.56 and 24.70 percent, respectively on the mycelial growth of *P. aphanidermatum*, whereas, the pathogen was not inhibited on exposure to the volatiles of macro-basidiomycetes fungi. The volatiles of *T. asperellum* showed the maximum inhibitory effect of 69.26 percent against *P. aphanidermatum.* The study also included the identification of Volatile Organic Compounds (VOCs) involved in the suppression of pathogens by Headspace Gas Chromatography Mass Spectrometry (HS GCMS). The results revealed the production of carvone by the leaves of *M. spicata*; citronellol and geraniol by *C. citratus*; isopentyl alcohol and limonene by *T. asperellum* with increased peak area percentage and these compounds possessed antifungal properties. The vaporous action of isopentyl alcohol completely suppressed the mycelial growth of *P. aphanidermatum*, which is highly correlated to the *T. asperellum* extract on pathogenic growth. While the compounds, carvone, and citronellol showed the maximum inhibitory effect of 89.02 and 85.49 percent, respectively when used at 500 ppm and also altered the sporulation behavior of *P. aphanidermatum.*

**Conclusion:**

Results showed that volatiles of *M. spicata* and *T. asperellum* have anti-oomycetes action on pathogenic growth leading to a distortion of sporulation of *P. aphanidermatum*. High antifungal properties make VOCs suitable for incorporation as a new integrated plant disease management programs.

**Supplementary Information:**

The online version contains supplementary material available at 10.1186/s12870-023-04351-3.

## Background

Crop plant infected by the plant pathogen under field and storage conditions is a major concern of economically important yield losses across the globe and also indirectly poison human with fungal toxin [1, 2]. Soil-borne plant pathogens are a major threatening disease that infects a vegetable crop from the seed emergence stage to the fruiting stage and sometimes during post-fruiting stages also. A broad species of *Fusarium* sp., *Verticillium* sp., *Sclerotinia* sp., *Pythium* sp., and *Phytophthora* sp. caused by vascular wilt, damping off, and seedling root rot disease and showed abundant loss to the tomato production [3, 4]. Among the soil-borne fungal pathogens, Damping off caused by *P. aphanidermatum* (Edson) Fitz. infect tomato crops from seedling to flowering stage, spreads through the soil, and enter root into the hypocotyl region causing seedling death and toppling down symptoms under nursery and field conditions, respectively. Further, considerable tomato production was reduced due to the abundant presence of pathogen propagules surviving in the soil [5, 6]. The agricultural practices in the current generation highly depend on the use of agrochemicals to protect crops from plant pathogens and also create low negative impact on the environment. The botanical and microbial origin has been driven to develop new organic products that are effective, and safer than current chemical fungicides [7]. To minimize the use of synthetic chemical fungicides, the studies on volatile organic compounds (VOCs) creates greater attention to discover new natural product for plant disease management. Volatile compounds have recently become more popular as potential BCAs as similar to endo-microbiomes.

VOCs are low molecular weight (< 300 Da) organic compounds that diffuse the volatiles at high vapor pressure with low polarity [8]. Earlier, scientists have identified and characterized diverse volatile chemical compounds emitted by natural plants and microbes that act as antagonistic agents [9]. Plant and microbes induced VOCs are chemically diverse and include alkenes, alcohols, ketones, terpenes, benzenoids, aldehydes, pyrazines, acids, esters, and sulfur-containing compounds. VOCs act as signalling molecules for both intra- and interspecific communication and possess potent antimicrobial agents. However, the interaction between the volatiles and the pathogen remains unknown [10].

In recent generations, the research on the volatile interactions between plants and pathogens becoming more potent in the suppression of plant pathogens by the use of botanicals, beneficial microbes, and endophytic microorganisms, and is known to have diverse antimicrobial properties [11]. In a few research studies, volatiles from essential oils obtained greater attention rather than natural sources. Specific volatile compounds induced by plant extracts or essential oils were analyzed for fumigation [12]. Scientists [13] reported that the volatiles from the beneficial microbe, *Pseudomonas aeruginosa* PC5 inhibited the fungal growth of *P. aphanidermatum* and their suppression reveals the production of dimethyl disulfide as a major component. Similarly, the research on volatile studies reporting still now, but their biochemical reaction and sporulation activity remains unknown. This study aims to evaluate the anti-oomycetes activities of natural plants and microbes against the fungal growth of damping-off caused by *P. aphanidermatum*.

## Results

### Isolation of *P. aphanidermatum* and molecular characterization of pathogen

**Fig. 1:**
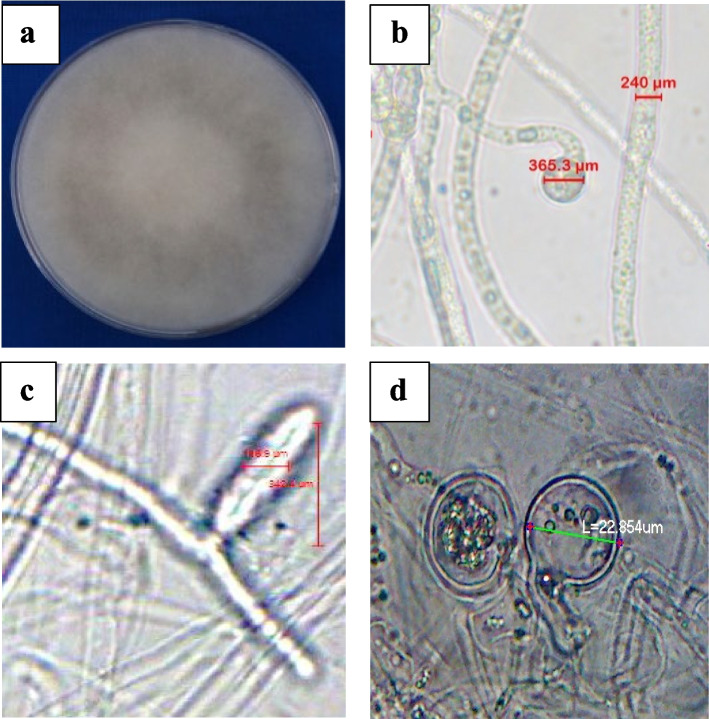
Morphological characteristics of *P. aphanidermatum.***a**. Fungal culture of *P. aphanidermatum;***b**. Aseptate mycelium; c. Sporangia and d. Oospore

The isolated fungus associated with the damping-off of tomato showed colorless, thick mycelia with cottony growth within two days of incubation. The morphological characters of *Pythium* culture were observed under a phase contrast microscope (200x) which revealed hyaline aseptate hypha (width ranged from 10 to 12 µm) with lobed sporangium (size varied from 39 to 43 µm in length and 20 µm in width). The sexual organs produced were terminal oogonia which appeared globose to spherical with a smooth wall (size: 21–24 µm). The male gametangia were antheridia found intercalary. The antheridium (10–12 µm long and 11–14 µm wide) pierced oogonium and produced an aplerotic oospore (size: 23–26 µm wide) with 1–2 µm thickness (Fig. 1). The morphometric observation of the isolated pathogen confirmed the identity of *Pythium aphanidermatum.* Amplification of DNA of the culture was done using ITS 1 and ITS 4 primers and the amplicon size was ~ 830 bp. The amplified sequence in comparison with the NCBI database showed 99 percent similarity with *P. aphanidermatum*. The sequence was submitted to the NCBI database and assigned with Genbank accession no: MW350044.

### Screening of natural plants and microbes for Antagonistic activity

**Fig. 2:**
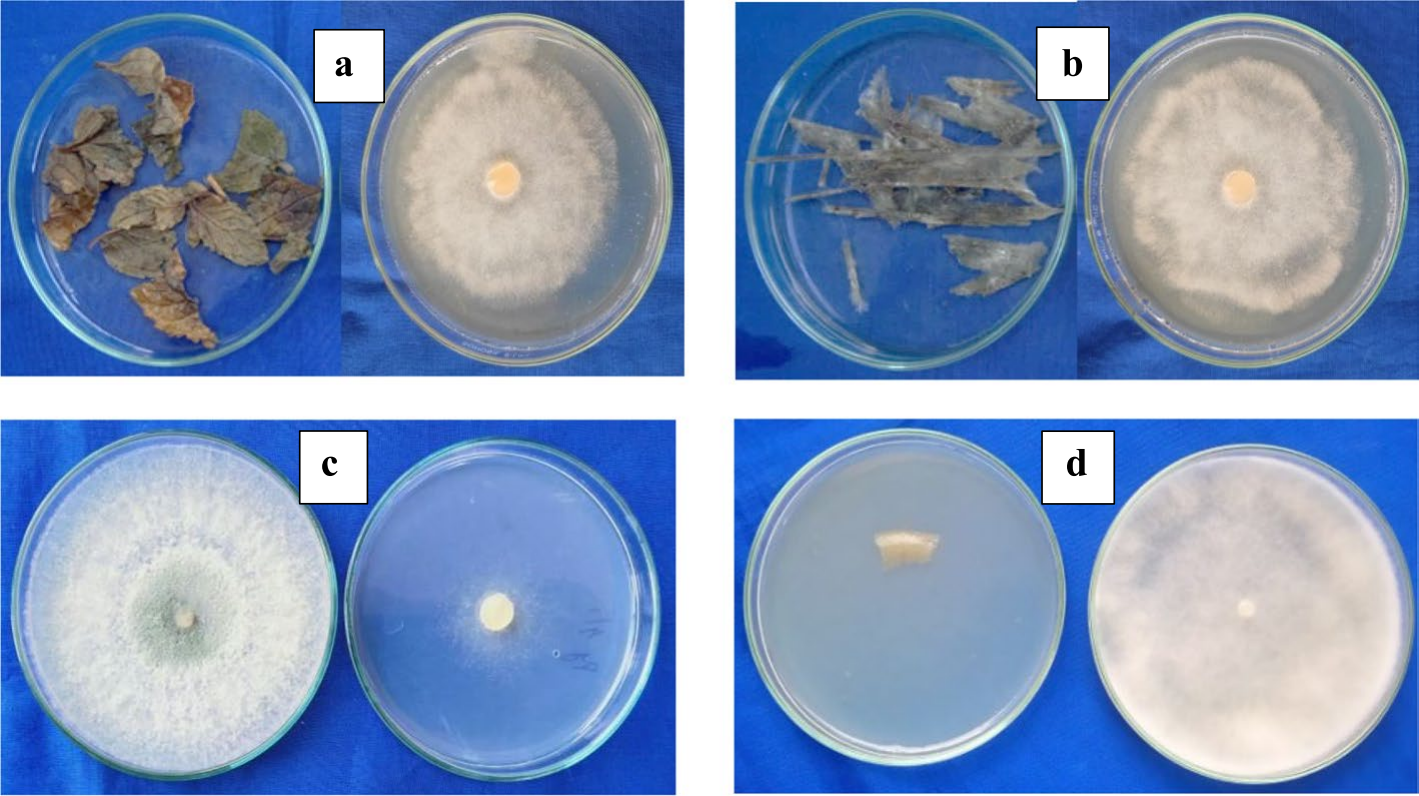
In vitro assessment of plant and microbial volatilomes against *P. aphanidermatum.* The volatiles of *M. spicata* (**a**), *C. citratus* (**b**) and *T. asperellum* (**c**) against pathogen (Control – (**d**))

The volatiles released by the leaf samples of *M. spicata* inhibited *P. aphanidermatum* to an extent of 45.56 percent as compared to the control followed by leaf sample of *C. citratus* (24.70 percent) and *V. negundo* (18.88 percent) (Fig. 2). The volatilomes from the leaves of *C. amboinicus* showed the least inhibition (3.70 percent), while the leaf samples of *V. zizanioides, O. tuniflorum,* and *A. indica* were ineffective. The mycelial characters and colony morphology of *P. aphanidermatum* were much altered due to the exposure of volatiles, wherein the mycelia became thin; sparse, and fragile with lesser growth as against the profuse normal growth (width 10–12 µm) in the control plate. The sexual and asexual spores of *P. aphanidermatum* were absent when exposed to the volatiles produced by *M. spicata* and *C. citratus* leaves (Figure S1; Table S1).

**Fig. 3:**
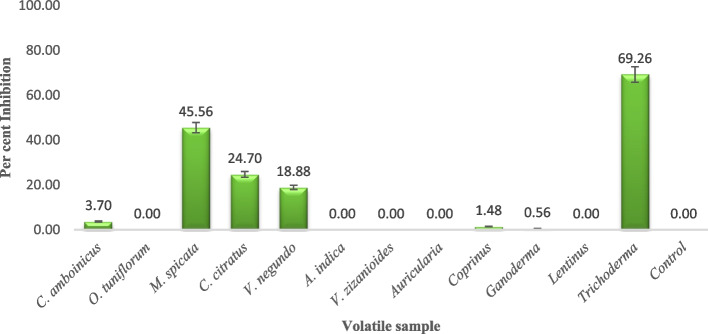
Screening of plant and microbial volatiles against *P. aphanidermatum.* Data are the mean of four replications and repeated thrice

In addition, the volatiles produced by the mycelia of *Auricularia auriculata, Coprinus cinereus, Ganoderma lucidum,* and *Lentinus edodus* did not show any inhibitory activity on the mycelial growth of *P. aphanidermatum* as compared to control plates. The volatiles from *T. asperellum* recorded the maximum inhibition of 69.26 percent with complete morphological alteration in the growth of the pathogen (poor in aerial hyphal growth and reduced in size with 2.3–2.8 µm width) compared to profuse colorless, thick cottony mycelial growth (12–12.5 µm width) in the control plates. The effect of volatilomes emitted from *B. subtilis* and *S. rochei* showed no inhibition on the mycelial growth of the pathogen (Fig. 3). Furthermore, sporangiospores, antheridia, and oogonia were found inhibited and their structural integrity was also found altered when exposed to the volatiles produced by the mycelia of *T. asperellum* (Table S2).

### Volatile profiling using HS GCMS

**Fig. 4:**
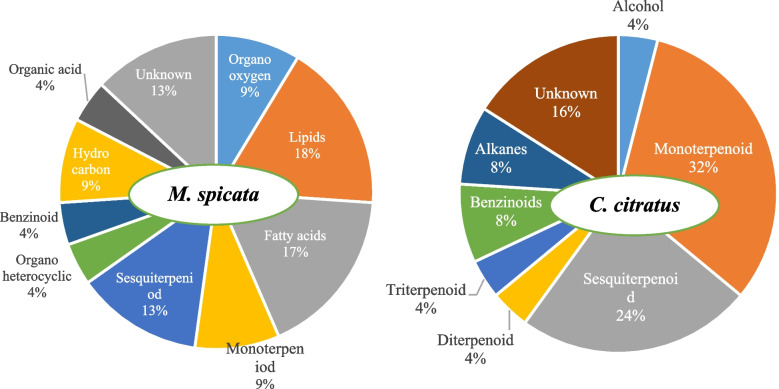
Classes of VOCs produced by plant samples

**Fig. 5:**
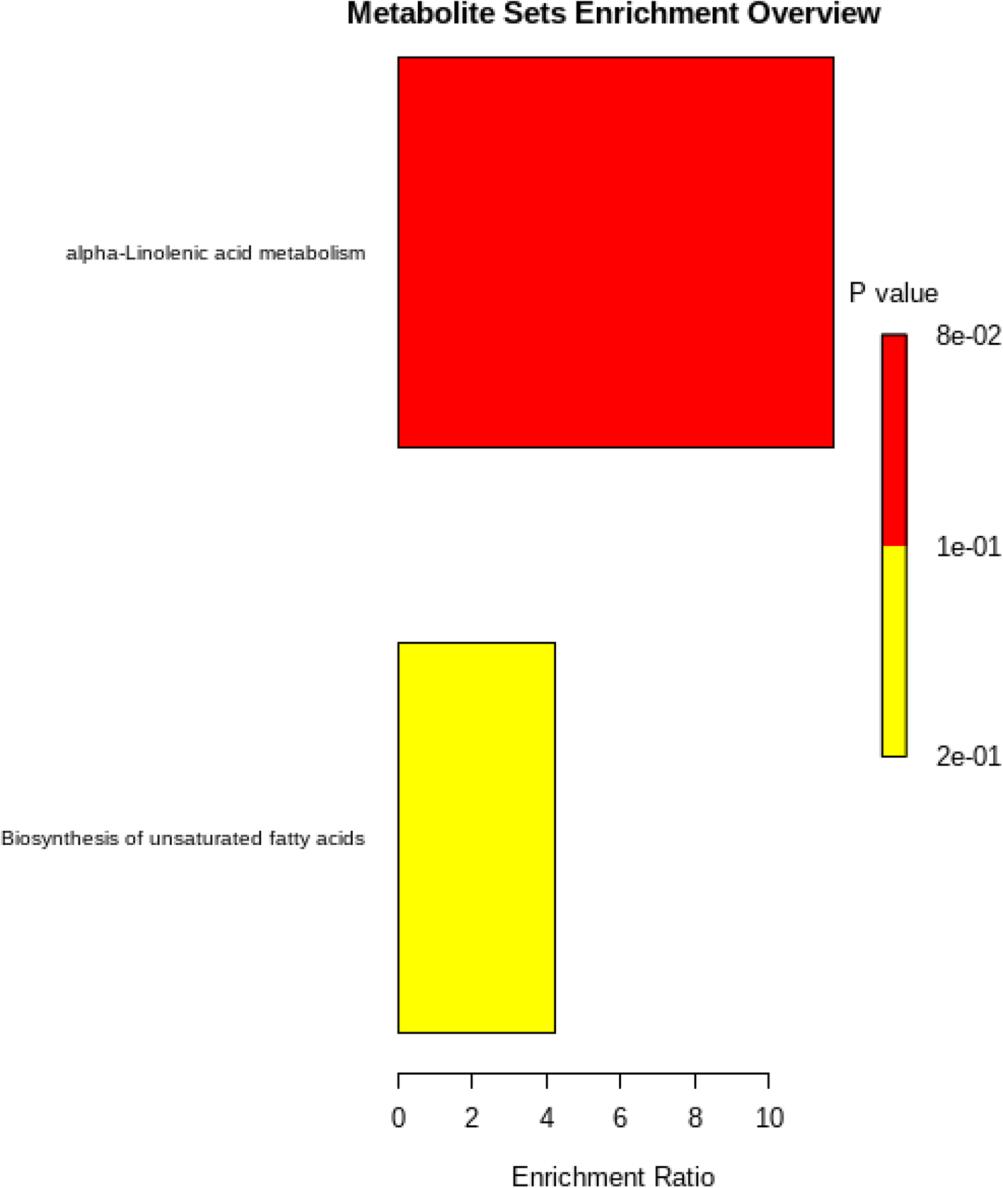
Pathway enrichment analysis of *M. spicata* and *C. citratus* VOCs

The result of HS GCMS revealed a total number of 25, 25, and 20 important volatile compounds produced by the leaves of *M. spicata, C. citratus,* and *V. negundo*, respectively. The compound, (-) -carvone was the most abundant VOC produced by *M. spicata* leaves with a higher relative abundance of 3.08 percent peak area at 10.25 RT (Table S3). Geraniol and citronellol were produced by *C. citratus* leaves with a high relative abundance of 15.85 and 5.27 percent peak area at 10.37 RT and 9.97 RT, respectively (Table S4). The VOCs from the leaves of *V. negundo* did not show a peak area of above 0.5 percent. The VOCs produced by *M. spicata,* and *C. citratus* belonged to varied classes of volatile metabolites as presented in Fig. 4. Over enrichment pathway analysis indicated that more than seventy volatile metabolites could also be profiled from the leaf samples of *M. spicata* and *C. citratus* regulated through alpha-linolenic acid metabolism and unsaturated fatty acids biosynthesis pathway (Fig. 5).

**Fig. 6:**
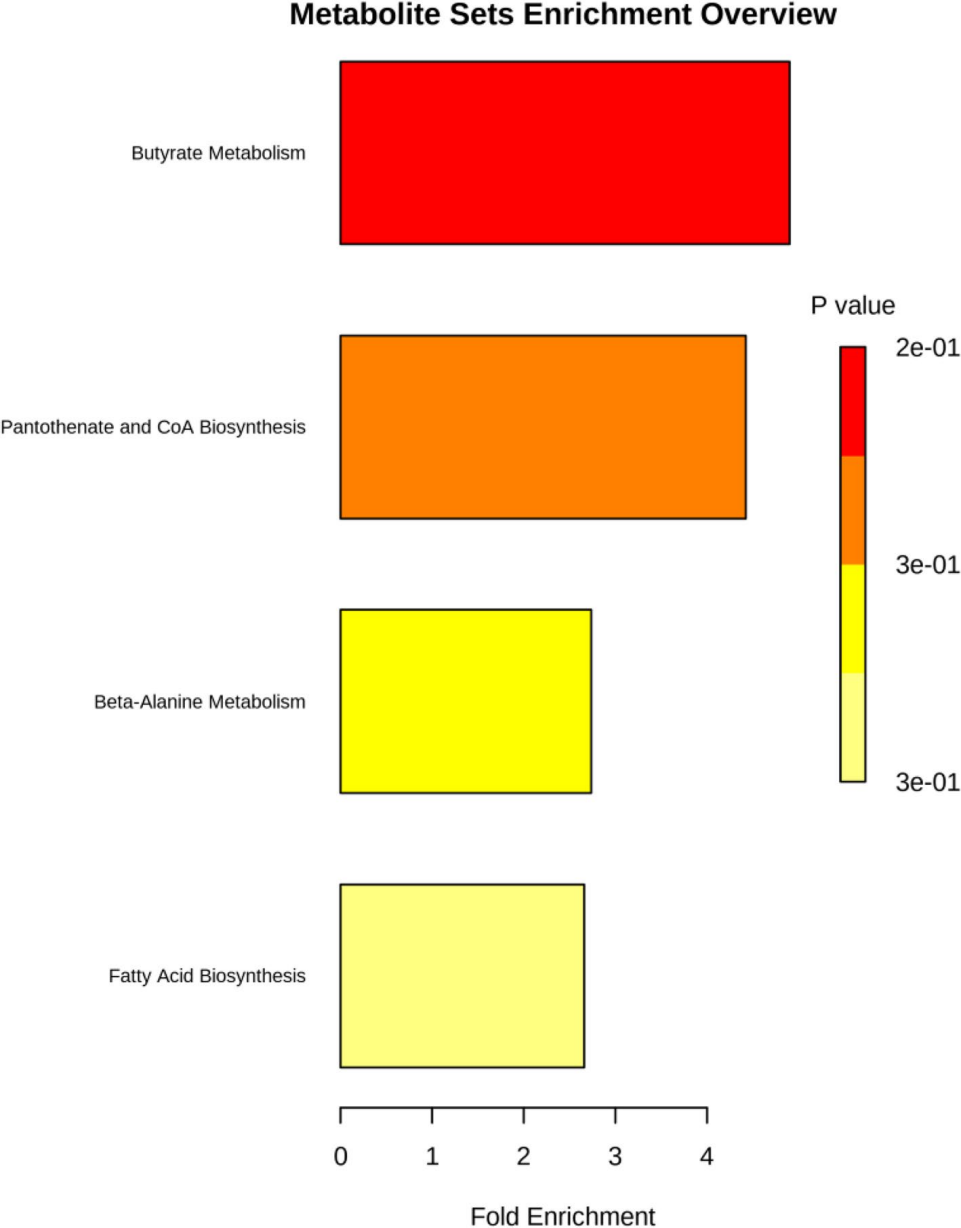
Pathway enrichment analysis of *T. asperellum* VOCs

The GC–MS-TD analysis of VOCs trapped by Tenax column from microbial cultures indicated that *T. asperellum* could produce diverse volatile compounds, including aldehydes, ketones, alcohols, esters, fatty acids, etc*.* Based on 90 percent similarity of mass spectra, a total number of 20 VOCs were obtained from the mycelia of *T. asperellum* (Table S5). Among these, isopentyl alcohol recorded a higher peak area of 3.4 percent (10.107 RT) followed by limonene (1.2 area percent; 12.27 RT). The other VOCs profiled were found to have low peak area abundance. These VOCs produced by *T. asperellum* were found to be associated with alcoholic compounds (25 percent), fatty acids, and prenol lipids (10 percent). The obtained volatile compounds were regulated by butyrate metabolism, pantothenate, and CoA biosynthesis, Beta-alanine metabolism, and fatty acids biosynthesis were conspicuous by over-enrichment pathway analysis (Fig. 6).

### Antagonistic activity of VOCs

**Fig. 7:**
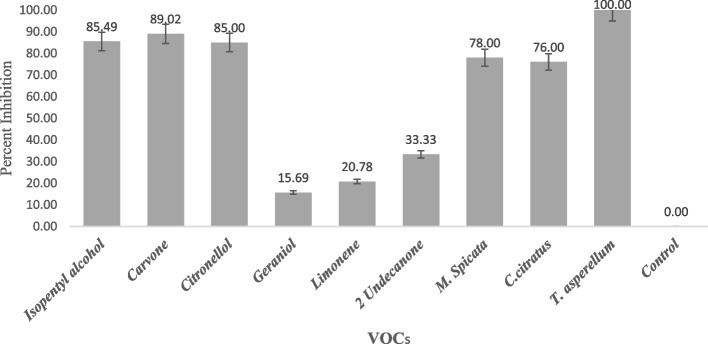
Antagonistic effect of pure VOCs and their volatile extract of *M. spicata, C. citratus and T. asperellum* against *P. aphanidermatum.* Data are the mean of four replications and repeated thrice

**Fig. 8:**
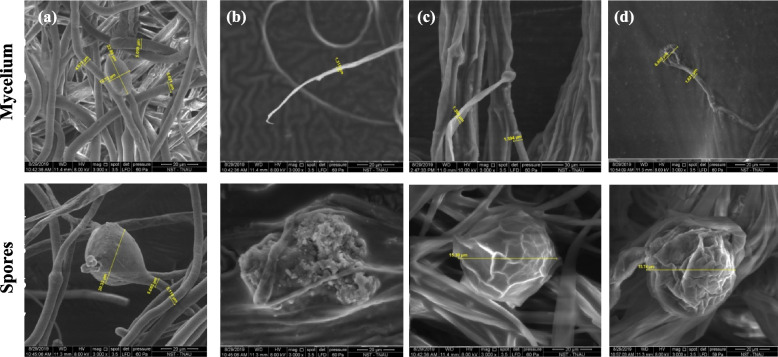
SEM images (3000 × magnification) of VOCs exposed mycelia of *P. aphanidermatum.* **a**. Non exposed mycelium and spore; **b**. *T. asperellum* volatile exposed; **c**. *M. spicata* volatile exposed and **d**. *C. citratus* volatile exposed

The standard VOCs such as the vapor phase of carvone, citronellol, geraniol, limonene, and isopentyl alcohol was used to test the anti-oomycete activities against *P. aphanidermatum* at five different concentrations (100, 200, 300, 400 and 500 ppm). The result of the antagonistic assay revealed that isopentyl alcohol flow throughout the compartment plate could completely suppress the mycelial growth of *P. aphanidermatum* at 500 ppm and the least inhibition level was recorded at 100 ppm. However, the mycelia of *P. aphanidermatum* did not express virulent growth in the bipartite plate exposed to VOCs. Carvone at 500 ppm also inhibited the mycelial growth of *P. aphanidermatum* up to 89.02 percent (Fig. 7). Colorless, stranded, sparse, floccose, and depressed mycelial growth with reduced size of mycelium was noticed with carvone volatiles exposure as compared to colorless, thick cottony mycelial growth in control plates. The volatiles of citronellol recorded 85 percent inhibition of mycelia at 500 ppm and the hyphae were lacking, while they were fresh, white, and thick cottony growth in comparison with that of the control plates. The least inhibition of mycelium was noticed with the volatiles of limonene (20.78 percent) and geranial (15.69 percent) at 500 ppm. However, it was also noticed with these treatments that there was also a conspicuous suppression of mycelial growth with thin irregular sectors of growth in comparison to that of the normal profuse mycelial growth in control. The hexane-extracted volatiles of *T. asperellum* also completely inhibited the mycelial growth of *P. aphanidermatum* in bipartite plates (100 percent) following the hexane-extracted volatilomes of *M. spicata* and *C. citratus* (up to 79 percent). The mycelia of *P. aphanidermatum* were also found to be completely shrunk, densely sparse with floccose in appearance (Fig. 8).

### Antifungal activity on sporulation of pathogen

The structural and morphological changes of mycelium, sexual and asexual spores of *P. aphanidermatum* exposed to the volatiles of *M. spicata, C. citratus* and *T. asperellum* were subjected to SEM. The SEM photographs at 3000 × magnification displayed that the mycelia of *P. aphanidermatum* were severely altered and the hyphae of pathogens were found to be distorted completely. The mycelia were diverged and flattened with retreated cell contents. The sexual spores of pathogens were irregular in shape, and malformed when exposed to a 500 ppm concentration of the volatiles of *T. asperellum* (Fig. 8). The mycelia of *P. aphanidermatum* exhibit convoluted and reduced hyphal growth with 2.67 µm width due to the activity of *M. spicata* and the sporangia were conspicuously fragile (9.23 µm diameter). Based on these SEM images, it is evident that the volatiles of *T. asperellum* and the vapour phase of isopentyl alcohol could heavily contort the mycelial growth and sporulation of *P. aphanidermatum*.

### Spore inhibition assay

**Fig. 9:**
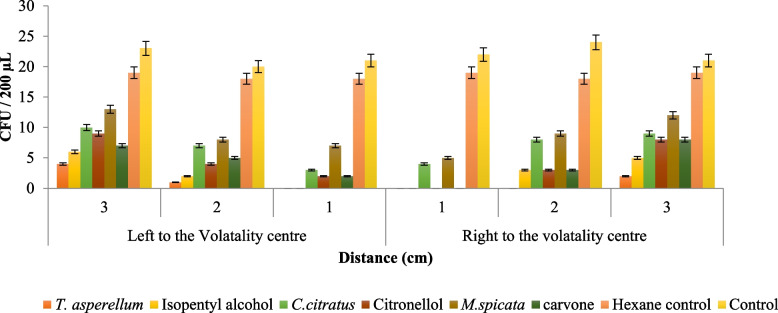
Vaporous activity of volatilomes on spore inhibition of *P. aphanidermatum* using 96 well microtiter plates. Data are the mean of four replication repeated twice

The zoospores of *P. aphanidermatum* were found to be significantly inhibited (0.00 OD) in the nearest wells (one cm away) to the volatility center. The inhibition percentage was comparatively less (0.16 OD) at a distance of 2 cm and the least percentage (0.37 OD) of inhibition was noticed in wells at a 3 cm distance when Isopentyl alcohol was used. The hexane extract of *T. asperellum* showed similar inhibitory action (0.01 OD; 150 min) when compared to standard volatiles of isopentyl alcohol (0.00 OD; 150 min). The volatiles of hexane extract of *C. citratus* and citronellol exposed plates showed similar inhibition with 0.3 OD on 150 min. VOCs of carvone showed maximum inhibition of zoospores (0.04 OD; 150 min), however, the hexane extract of *M. spicata* did not show the best OD value as it crosses above 0.35 OD. The maximum OD value was shown during 0, 30, 60, 90, and 120 min, which indicates that the volatile compound did not show any inhibitory action on spore germination as in control (0.42 OD; 150 min) and hexane control (0.40 OD; 150 min) exposed plates. Later, the suspended spore inoculum was pipetted out after periodical observation and resuspended in a PDA medium to find out the percent inhibition in the colony-forming unit (cfu). The standard compound isopentyl alcohol and volatilomes of *T. asperrellum* exposed plates completely suppressed the spore germination of *P. aphanidermatum* in the nearest wells (one cm distance well) from the volatility center. From the present observation, volatilomes extract of *T. asperrellum* exposed micro-titer plates were found to have a fungicidal reaction at one cm distance wells, while they showed fungistatic reaction at 2 and 3 cm distance wells away from the volatility center (Fig. 9).

## Discussion

Volatilomes mediated plant defense could provide a promising outcome for plant disease management and growth promotion. These volatilomes are considered as info chemicals on the interaction between the plants and pathogens [14]. The plant volatiles travels long distances and provides extreme variation in their biological properties and this creates a curiosity challenge among the investigators to analyze the volatiles produced by certain plants and explore their biosynthetic pathway [15]. The present study focused to determine the potential of volatiles produced by plants and microbial organisms against damping off causing pathogens and understanding the tripartite interaction between the volatilomes, respective pathogens, and tomato plants to reveal the actual defense mechanism. Damping off symptoms were expressed in the tomato seedlings as a water-soaked lesion at the collar region, rotting, and wilting of plants. In the present study, the isolated damping-off pathogen produced white, thick cottony profused aerial mycelium on PDA media; which is similarly observed by past researchers [16–18].

The volatilomes produced by the leaves of *M. spicata* inhibited *P. aphanidermatum* (45.56 percent) with several morphological alterations on the mycelial growth and also inhibited the spore germination of the pathogens. The volatilomes produced by the leaves of *C. citratus* reduced the mycelial growth by 24.78 percent fungal growth of *P. aphanidermatum*. This is in correlation with earlier reports [19] that the volatiles of medicinal plants had inhibited the mycelial growth of *Penicillium digitatum, Didymella bryoniae, Colletotrichum lindemuthianum, Fusarium solani, Rhizoctonia solani,* and *Pythium ultimum* and also caused certain morphological changes on exposure to the volatiles.

In addition to the present finding, several microbial cultures are known to produce a wide variety of antimicrobial substances naturally, active against a large spectrum of phytopathogenic microorganisms [20–22]. Based on these facts, the current study experimented with the antifungal volatile activities of selected microbes against *P. aphanidermatum*. The volatiles produced by the mycelia of *A. auriculata, C. cinereus, G. lucidum,* and *L. edodus* did not show any inhibitory potential on the growth of *P. aphanidermatum*. But, the effect of *T. asperellum* volatiles exerted 69 percent of mycelial growth inhibition on *P. aphanidermatum* using inverted plate assay. Hence, the antifungal nature of *T. asperellum* may be attributed to the production of volatiles. The flow of microbial volatiles throughout the plates could have significant antagonistic activity. Besides mycelial growth inhibition, the negative effects on sporulation of *P. aphanidermatum* were also noticed with distorted appearance as reported earlier [23]. Other findings also reported that the volatiles from different isolates of *Trichoderma* had antifungal and antibacterial activities against plant pathogens [24, 25]. The present study hypothesizes that the indirect interaction of antifungal activity of volatiles produced by microbes might be responsible for pathogen inhibition.

In order to interpret the nature of VOCs produced by *M. spicata* and *C. citratus* leaves, HS coupled with GC–MS was performed to trace VOCs even at a lower concentration through a headspace analysis. Scientists [26] revealed on the use of air entrainment techniques to trap volatiles emitted by the adult insect (*Conogethes punctiferalis*) from the sample chamber. In this study, the volatiles trapped in fresh leaves of *M. spicata* and *C. citratus* were further subjected to GC–MS analysis. The relative percentage of compounds present was detected in the headspace analysis. The important VOCs produced by the leaves of *M. spicata* and *C. citratus* leaves were identified as carvone, citronellol, and geraniol, respectively. VOCs produced by the leaves of *M. spicata* and *C. citratus* read through MS spectral library strongly supported the earlier results [27–30] who have documented matching VOCs profile with various species of *M. spicata* and *C. citratus*. Similarly, 20 important volatile compounds were detected from the mycelial growth of *T. asperellum*. Among the 20 compounds, isopentyl alcohol and limonene were the most abundant VOCs, which secured high peak area percent which highly correlated with the present findings [31] of *T. harzianum, T. virens,* and *T. atroviride* volatile compounds. The standard volatile compounds were used to determine the best concentration against the target pathogens. *In-vitro* experiments revealed that carvone and citronellol had effectively inhibited the mycelial growth of *P. aphanidermatum* and brought out abnormalities in the mycelia of pathogens at the concentration of 500 ppm and possessed broad spectrum anti-oomycetes properties. Similar experimental analyses [32] have been reported with a large group of fungal pathogens namely, *Alternaria alternata, A. solani, Fusarium acuminatum, F. chlamydosporum, F. culmorum, F. equiseti, F. graminearum, F. incarnatum, F. nivale, F. oxysporum, F. proliferatum, F. sambucinum F. scirpi, F. semitectum, F. solani, F. tabacinum, F. verticillioides, Pythium ultimum* and *Rhizoctonia solani*. The least inhibition was shown at the lowest concentration of volatiles (100 ppm). The SEM study also proved that the hyphal growth of the pathogens was reduced in size and the spores were completely distorted. The compound, Isopentyl alcohol produced by the mycelia of *T. asperellum* exhibited significant inhibition of mycelial growth of *P*. *aphanidermatum* to a maximum of 100 percent when exposed to 500 ppm concentration. Earlier studies proved with these research that the VOCs of *Trichoderma harzianum* inhibited the mycelial growth of soil-borne pathogens [33, 34]. The volatiles of *T. asperellum* suppressed the mycelial growth, which showed weak, shrunken and finally kills the mycelial growth of the pathogens.

The vapor phase of isopentyl alcohol completely suppressed the spore germination of *P. aphanidermatum* in the 96-well microtitre plates. This is in agreement with the earlier findings [35]. The vapourous phase of isopentyl alcohol also acts as a fungicidal effect on spore germination of *P. aphanidermatum* rather than the hexane extract of *T. asperellum* volatiles. Likewise, the natural extract of *Combretum racemosum* volatiles had fungicidal activities toward the colony growth of *Pythium aphanidermatum* [36]. All these findings support the logic of the present findings. Our experimental analysis clearly demonstrated the highly toxic activities of isopentyl alcohol suppresses the nature of fungal growth of *P. aphanidermatum* in comparison to other VOCs.

## Conclusion

Our study concluded that volatiles from *T. asperellum, M. spicata,* and *C. citratus* was shown to exhibit a maximum level of pathogen inhibition. VOCs of Isopentyl alcohol, carvone, and citronellol were characterized by trapping the volatilomes and also assessed as a major compound for the suppression of *P. aphanidermatum*. It is clear that volatiles from natural plants and microbes have rich sources of VOCs, which possess anti-oomycetes activity against *P. aphanidermatum*. High volatile properties make the plant and microbes induced compounds suitable for the development of new integrated environment friendly disease management strategies. These results show that application of volatiles from natural plants and beneficial microbes could have a toxic effect on the fungal growth of plant pathogens.

## Methods

### Plant material and microbial culture collection

Medicinal herbs such as mint (*Mentha spicata* L.), lemon grass (*Cymbopogon citratus* DC.), coleus (*Coleus amboinicus* L.), white chaste leaf (*Vitex negundo* L.), holy basil (*Ocimum tenuiflorum* L.), neem (*Azadirachta indica* A.) and vetiver (*Vetiveria zizanioides* L.) were collected from the Department of medicinal and aromatic plants, Tamil Nadu Agricultural University, Coimbatore, India. The macro-basidiomycetous fungi such as *Ganoderma lucidum* (Curtis) (MN729467), *Auricularia auriculata* (Bull.) (MN729469), *Lentinus edodus* (Berk.) (MN729468), *Coprinus cinereus* (Schaeff) (MH 444367), *Trichoderma asperellum* (KJ803854), *Bacillus subtilis* (KJ603239) and *Streptomyces rochei* (MN631088) were obtained from the TNAU Bio-Resource Centre, Department of Plant Pathology, TNAU, Coimbatore. The raw materials were stored in a sealed polybag for the experimental analysis.

### Symptom collection, isolation, and a confirmatory assay of the pathogen

Plant samples showing damping off symptoms were collected from the infected tomato nurseries. The pathogen was isolated from the collar region of the infected sample which was surface sterilized using 0.1% sodium hypochlorite solution. Then the sterilized infected tissue was placed on a sterile Petri dish containing the PDA medium and wrapped with cling film, incubated at room temperature (28 ± 2 °C) for mycelial growth. The freshly growing hyphal tip was spotted and plugged out with a 5 mm sterile cork borer and transferred to the fresh PDA medium in a Petri plate or test tube containing PDA medium for further studies. The pathogen was further identified by recording the morphometric characters of mycelium, sporangium, and oospores under a phase contrast microscope (Leica DM 2000 LED, Switzerland). The isolated fungi from the infected tomato plants were further subjected to molecular characterization to confirm the identity at the species level. Total genomic DNA from the mycelium was extracted using cetyl trimethyl ammonium bromide (CTAB) buffer as described earlier [37, 38].The quality of extracted DNA was checked using nanodrop and further confirmed by agarose gel electrophoresis. PCR amplification was performed using Emerald Amp® GT PCR master mix in a total volume of 10 µL reaction. The primers ITS 1 and ITS 4 were used for the amplification of internal transcribed regions of ribosomal DNA. The PCR reaction was carried out in a 96-well Thermal cycler (Applied biosystem, Thermo-Fisher Scientific, Thermal cycler, Foster city, CA) by following different cycling conditions: 94 °C for 5 min; 36 cycles of denaturation at 95 °C for 1 min, annealing at 54 °C for 30 s., extension at 72 °C for 80 s. and final extension at 72 °C for 10 min. The amplified PCR product was electrophoretically separated on one percent agarose gel electrophoresis with 2 µL of 100 kb ladder. The amplicon size of the PCR product was examined under the Bio-Rad gel documentation unit (UVITEC, Cambridge). The DNA sequences in Fasta format were obtained in a clear chromatogram. These sequences were entered in query sequences available in the nucleotide blast analysis program at the NCBI database. On retrieving the output data, the organism showing higher similarity was considered a closely related species. These determined sequences were submitted to the NCBI database for obtaining accession numbers.

### Volatility assay

The fresh leaves of mint, lemon grass, coleus, white chaste leaf, holy basil, neem, and vetiver were used for testing the antifungal activities against *P. aphanidermatum* using the sealed plate method. The blend of volatiles emitted from the medicinal herbs was also tested for efficacy by placing the crushed and mixed plant samples (one gram) in the bottom Petri plate. In the case of fungi viz., *A. auriculata, C. cinereus, G. lucidum, L. edodus, T. asperellum*; bacteria, *B. subtilis* and *actinobacteria*, *S. rochei* were used in this study. The mycelial plugs (5 mm) of fungi used were individually placed on a PDA medium at the center of a Petri dish. *B. subtilis* and *S. rochei* were separately streaked on the entire Petri dish containing NA medium. One 5 mm mycelial disc from 7-day old culture of *P. aphanidermatum* was placed at the center of a Petri dish containing a PDA medium. This plate was placed inverted on the other bottom plate, tightly sealed with cling film and completely wrapped with foil to prevent the diffusion of volatiles. The control plate was maintained in the same way without medicinal herbs. The Petri dishes were incubated at 28 ± 2 °C until the mycelia completely occupy the control plates. The alterations in mycelia's morphological characteristics and pathogens' reproductive structures were observed. Percent inhibition of fungal growth over control was calculated by using the formula [39]:

$$Per cent inhibition of fungal growth \left(PI\right)= C-T /C x 100$$

where C is the radial coverage of the pathogen in control, and T is the fungal growth of the pathogen in treatment. The experiment was performed thrice with three replications to confirm the efficacy of volatiles produced by medicinal herbs and microbes.

#### Plant volatilomes trapping assay

The volatilomes produced by the leaves of plant samples were trapped and analyzed using air- entrainment technique with slight modification [40]. The freshly crushed leaves were placed into the volatile chamber and tightly closed with a lid. The lid contained a hole inserted with a glass tube (outlet) for trapping the volatiles emitted by the samples. Another hole in the lid inserted with a glass tube (inlet) helped to pass moistened air inside the chamber. The inlet was connected to a fish tank motor pump to pass purified moistened air inside the volatile chamber through activated charcoal. The headspace (HS) volatiles were trapped using Poropak Q, a glass cartridge fitted on the outlet hole. The experiment was performed for 5–6 days to trap the volatiles emitted by the leaves of plant samples. Then, the trapped volatiles was gently eluted by adding 200 µL of HPLC grade hexanal up to 1 mL by volume and collected in a GC vial for identification of VOCs produced by the leaves of respective plant samples.

The trapped volatiles were immediately subjected to analysis by HS-GCMS using a Thermo GC injector coupled with Mass Spectrophotometer (Turbo Matrix 150, purchased from Perkin Elmer, USA). Helium was used as carrier gas (1.1 mL /min). The energy of electron impact was 70 eV, and the ion source and quadrupole temperatures were set up at 230 °C and 150 °C, respectively. Electron impact (EI) of mass spectra was programmed from the range of 20–220 atomic mass units at sec intervals. One microlitre of eluted volatiles from the sample was taken as an injector. The MS spectrum was programmed to detect the compounds based on the breakage of different ions. The important VOCs produced were shortlisted based on the percent area and probability of compounds shown in the mass spectrum. The spectrum of selected VOC was compared with the NIST MS version 2.2 data library for identification of the exact volatile compound produced by the sample.

#### Microbial volatilomes trapping assay

The volatilomes produced by microbes were collected using the tenax column. The tenax column is a stainless tube containing TA (2,6-diphenyl-p-phenylene oxide) porous polymer (Perkin Elmer, HO244966) of 8.89 cm in length. The microbial cultures separately were inoculated into the conical flasks containing PDA medium, tightly closed with parafilm, and wrapped with aluminum foil. The volatiles produced in the headspace was trapped using a setup fitted to the top of the conical flask through a single-holed rubber cork at the center, used to insert the tenax column. Half the portion of the tenax column remained inside the conical flask for trapping the headspace volatiles, while two fourth portion of the tenax column was outside the flask. The trapped volatiles in the tenax column was analyzed in GC MS coupled with automated thermal desorption (Turbo Matrix 150; Perkin Elmer, USA). Capillary column is gas chromatography (GC) columns that contain stationary phase being packed into the cavity to analyze the volatile compounds. The detection and analysis of VOCs produced by microbial cultures were performed using the trap and purge method [41]. The tenax column with trapped volatiles was fitted to an automated thermal desorber and the volatiles from the column were passed to GC at varied oven temperatures ranging from 50 to 250 °C at an increasing indent of 10 °C per minute. Helium gas was used as the carrier at 1.42 kg/cm2 and the electron impact of spectra was maintained at 70 eV. The spectra of each VOC obtained from GC MS were searched and identified with the spectra of data in the NIST database version 2.2. Further, the volatile pathway enrichment analysis was performed to identify the biologically meaningful patterns of metabolic pathways. Hence, the analysis was implemented to understand the biosynthetic pathway of volatile compounds.

#### Testing the effect of synthetics compounds by bipartite plate assay

Based on the efficacy of selective VOCs obtained from medicinal herbs and microbial cultures, standard compounds viz., carvone (W224901), citronellol (CRM40469), geraniol (W250716), isopentyl alcohol (309,435), limonene (183,164), and 2 undecanone (W309303) were purchased from Sigma Aldrich and tested individually for their possible inhibitory effect on the mycelial growth of the test pathogens using bipartite plate assay. The sterile PDA medium was poured into one-half of the divided plate and the water agar medium was poured into the opposing sector. One five mm diameter mycelial disc of the test pathogen was placed on the PDA medium one cm away from the edge of the plate. Subsequently, the standard volatile compound was drenched on a 5 mm sterile filter paper disc and placed over the water agar medium in the opposite sector. A filter paper disc without the standard volatile compound was used as the control. The compound used for drenching the filter paper was prepared at different concentrations to identify the effective concentration against the pathogens. All the plates were incubated at 28 ± 2 °C for 7 days and the diametrical expansion of mycelial growth (in mm) of the pathogen was measured. The experiment was repeated thrice with three replications. The same method was used for testing the antifungal activity of volatiles extracted from medicinal herbs and microbial cultures against the test pathogens, separately. The antifungal activities of eluted volatiles were compared with the volatile compounds produced by the standard samples. Since hexane was used for eluting the volatile compounds from the samples, hexane alone was also tested separately for comparison.

### Scanning Electron Microscopy (SEM)

The interactions of pure volatile compounds with the mycelial growth of test pathogens were visualized through SEM (FEI Quanta 250, Netherland at 3000 × magnification). The inhibited mycelium was cut from the compartmental plate, the medium adhering to the mycelia was gently sliced with a sterilized scalpel and the sample was air-dried to remove excess moisture. Then, the sample was coated with a golden sputter and visualized using SEM.

### Spore inhibition assay

#### Preparation of inoculum

Well grown five days old *P. aphanidermatum* culture was flooded with sterile distilled water and incubated at 25 °C for 3 days with continuous illumination (1200 lx/ ft. candle) to enhance the sporangia production. Then, the zoospores were forced to be released by chilling the flooded cultures at 4 °C for one hour followed by keeping the culture at 25 °C for 30 min [42]. The released zoospores were collected from the flooded suspension by filtration with three to four layers of sterilized muslin cloth. The zoospores thus, harvested in the filtrate were diluted to a desired dilution for further study. A loop full of spores was collected and suspended in 1 × phosphate buffer solution (8 g sodium chloride, 0.2 g potassium chloride, 1.44 g of disodium hydrogen phosphate, 0.24 g potassium dihydrogen phosphate in 1000 mL of sterile distilled water). The cell density of the suspended solution was measured by optical density at 600 nm (OD600). Then, the suspended spores (5 mL) were mixed with 50 mL of Rosewell Park Memorial Institute-1640 medium (RPMI 1640, Sigma-Aldrich). The final inoculum was prepared as described by Clinical and Laboratory Standards Institute (CLSI) guidelines (Wayne, 2008).

#### Vapor phase mediated spore inhibition assay

The vapor phase-mediated spore inhibition assay (VMS) was performed in a 96-well polystyrene microtiter plate. In a standard design, 200 µL (5 × 103 cfu) of suspended inoculum was added to all the 96 wells in the microtiter plate, except for well D/E 6–7. The hexane-eluted volatile compounds sample was added to well D/E 6–7 (500 µL) and demarked as a volatile diffusion center. Whereas, the blank was maintained without the spore suspension to well A-H, 1, and 12; or a separate microtiter plate was used to serve as blank. The microtiter plates were covered with a lid; wrapped with aluminum foil and incubated at 28 ± 2 °C. The periodical observation of cell density in the wells was made at 0, 30, 60, 90, 120, and 150 min. After incubation for a specific period, the plates were measured at OD600 with a multi-well plate reader (Spectromax molecular device I3X, USA). Later, the suspended cell inoculum in the 96 well plates was individually pipetted out after the periodical observation and resuspended in a PDA medium by following the procedure of serial dilution method to find out the fungistatic or fungicidal reaction of volatile compounds on the sporulation of pathogen. However, on further incubation devoid of volatiles, the pathogen regained its colony growth suggesting only the fungistatic nature of the volatile compound; if the pathogen fails to regrowth the sporulation on the suspended media suggests the fungicidal action of volatile compounds against the pathogen.

### Statistical analysis

The statistical analysis of data is assessed using SPSS (Version 10.0). The significant differences between the treatments were validated using analysis of variance (ANOVA) and Duncan’s Multiple Range Test (DMRT) at a 5 percent significance level. The metabolic pathway was predicted in metaboanalyst 5.0 software.

### Supplementary Information

**Additional file 1: Fig. S1.** Image illustrating the changes in morphological characters of *Pythium aphanidermatum *due to *in vitro *exposure of plant and microbial volatiles. a- *M. spicata *exposed culture, b- *C. citratus *exposed culture, c- *T. asperellum *exposed culture, d- control (Pathogenic culture) and e- sporulation of *P. aphanidermatum*. **Fig. S2.** Volatile action of hexane extract of *T. asperellum* (a)*, M. spicata *(b)*, C. citratus *(c)*, *VOCs of isopentyl alcohol (d), carvone (e) and citronellol (f) on *P. aphanidermatum* in bipartition plate. The image (g) represent hexane control and (h) pathogen control. **Table S1. **Changes in the morphological characters of *Pythium aphanidermatum* due to *in vitro* exposure of plant volatilomes. **Table S2. **Changes in the morphological characters of *Pythium aphanidermatum* due to *in vitro* exposure of microbial volatilomes. **Table S3.** GC-MS profiling of VOCs produced by leaves of *Mentha spicata*. **Table S4.** GC-MS profiling of VOCs produced by leaves of *Cymbopogon citratus*. **Table S5.** GC-MS profiling and volatile composition of *T. asperellum*.

## Data Availability

Data/findings were included in this article and supplementary materials.
