# Supplementary material for: Activity of volatiles induced by microbes and natural plants stifled the growth of Pythium aphanidermatum - the damping off in Tomato
Source: BMC Plant Biol. 2023 Aug 10;23:384. doi: 10.1186/s12870-023-04351-3 (PMC10413521; doi:10.1186/s12870-023-04351-3)
Supplement: Supplementary file 1 — Additional file 1: Fig. S1. Image illustrating the changes in morphological characters of Pythium aphanidermatum due to in vitro exposure of plant and microbial volatiles. a- M. spicata exposed culture, b- C. citratus exposed culture, c- T. asperellum exposed culture, d- control (Pathogenic culture) and e- sporulation of P. aphanidermatum. Fig. S2. Volatile action of hexane extract of T. asperellum (a), M. spicata (b), C. citratus (c), VOCs of isopentyl alcohol (d), carvone (e) and citronellol (f) on P. aphanidermatum in bipartition plate. The image (g) represent hexane control and (h) pathogen control. Table S1. Changes in the morphological characters of Pythium aphanidermatum due to in vitro exposure of plant volatilomes. Table S2. Changes in the morphological characters of Pythium aphanidermatum due to in vitro exposure of microbial volatilomes. Table S3. GC-MS profiling of VOCs produced by leaves of Mentha spicata. Table S4. GC-MS profiling of VOCs produced by leaves of Cymbopogon citratus. Table S5. GC-MS profiling and volatile composition of T. asperellum. [file 12870_2023_4351_MOESM1_ESM.docx]

**Supplementary Material**

**Activity of volatiles induced by Microbes and Natural Plants stifled the growth of *Pythium aphanidermatum* - The Damping off in Tomato**


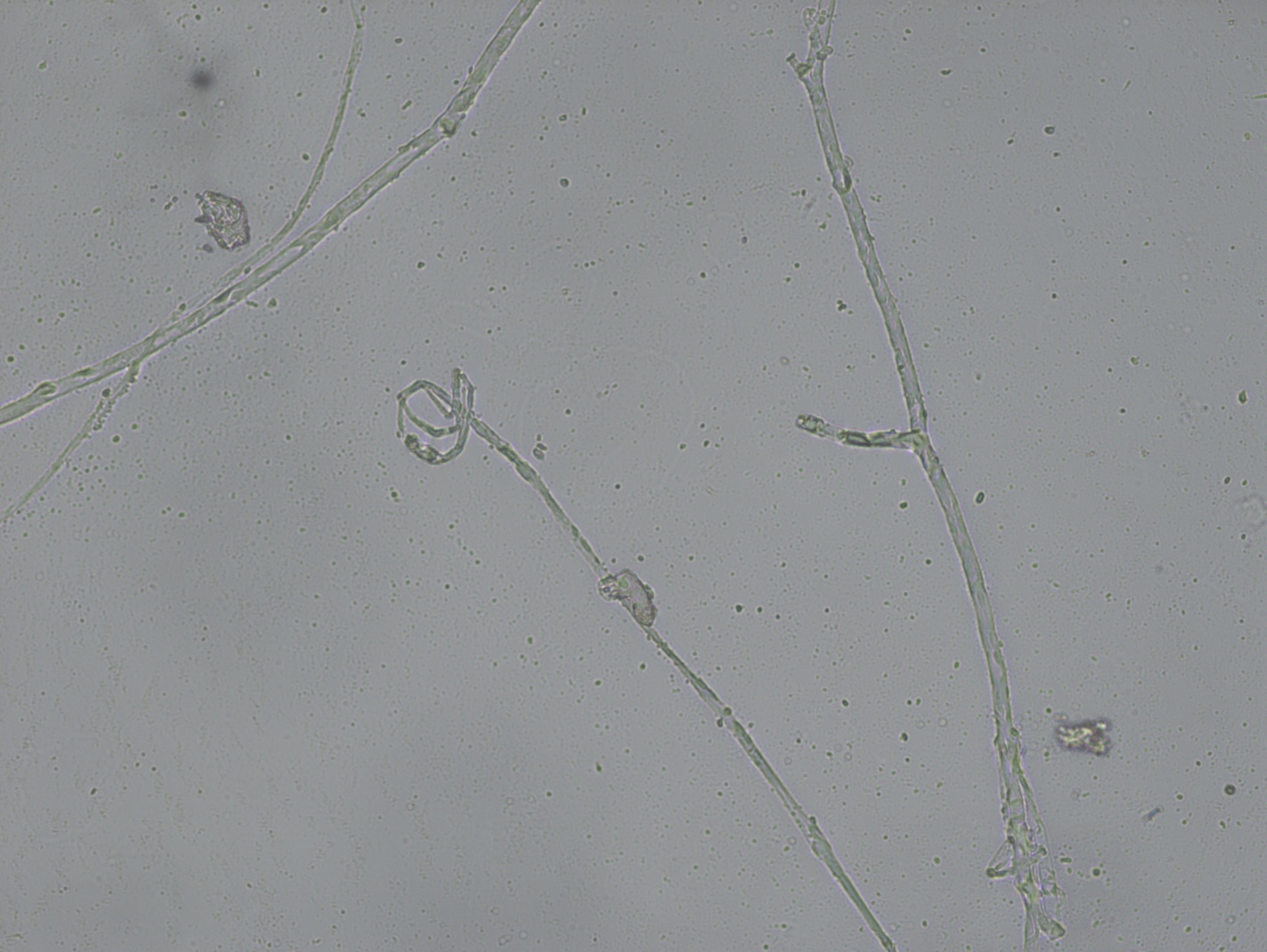

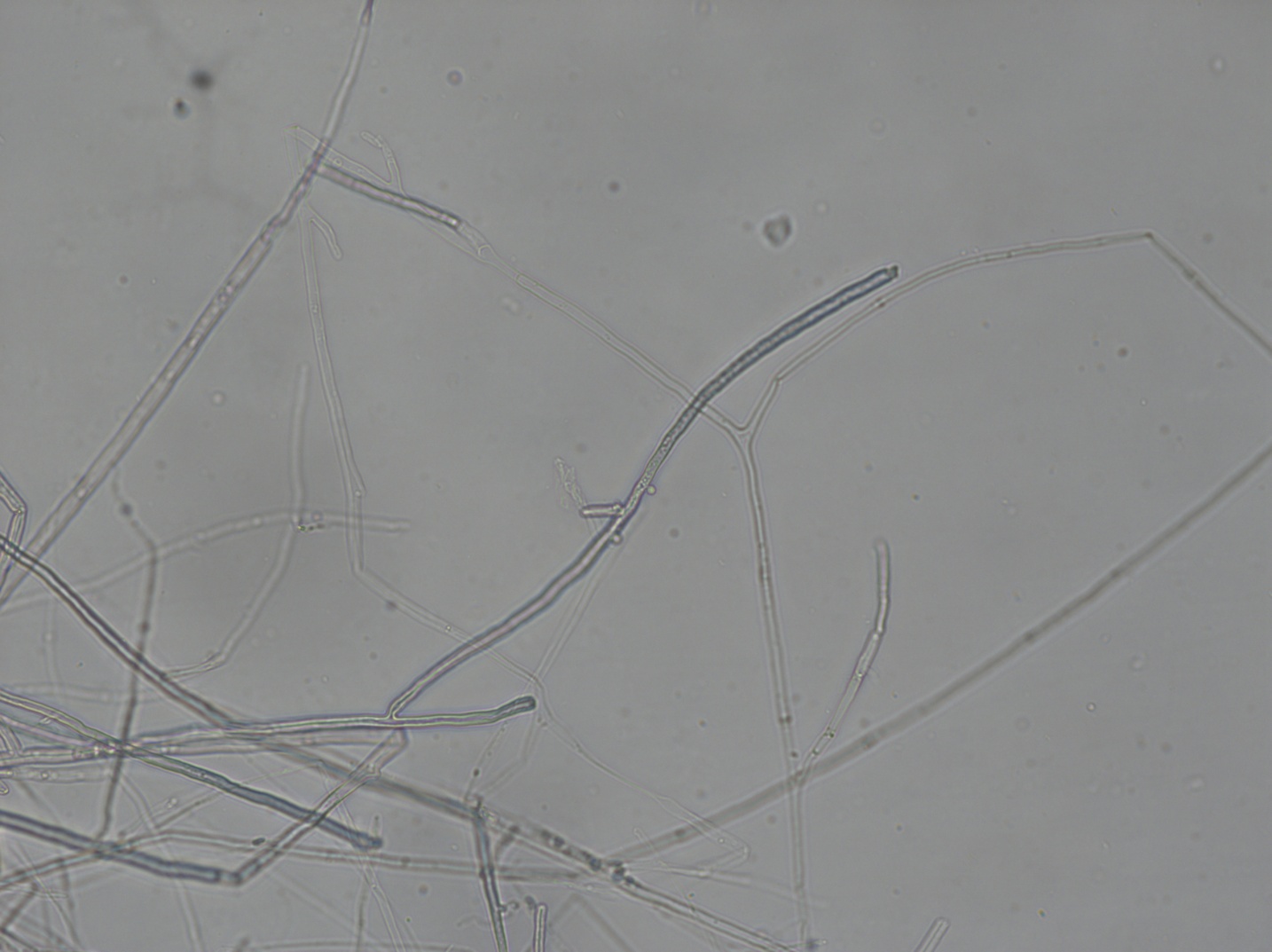

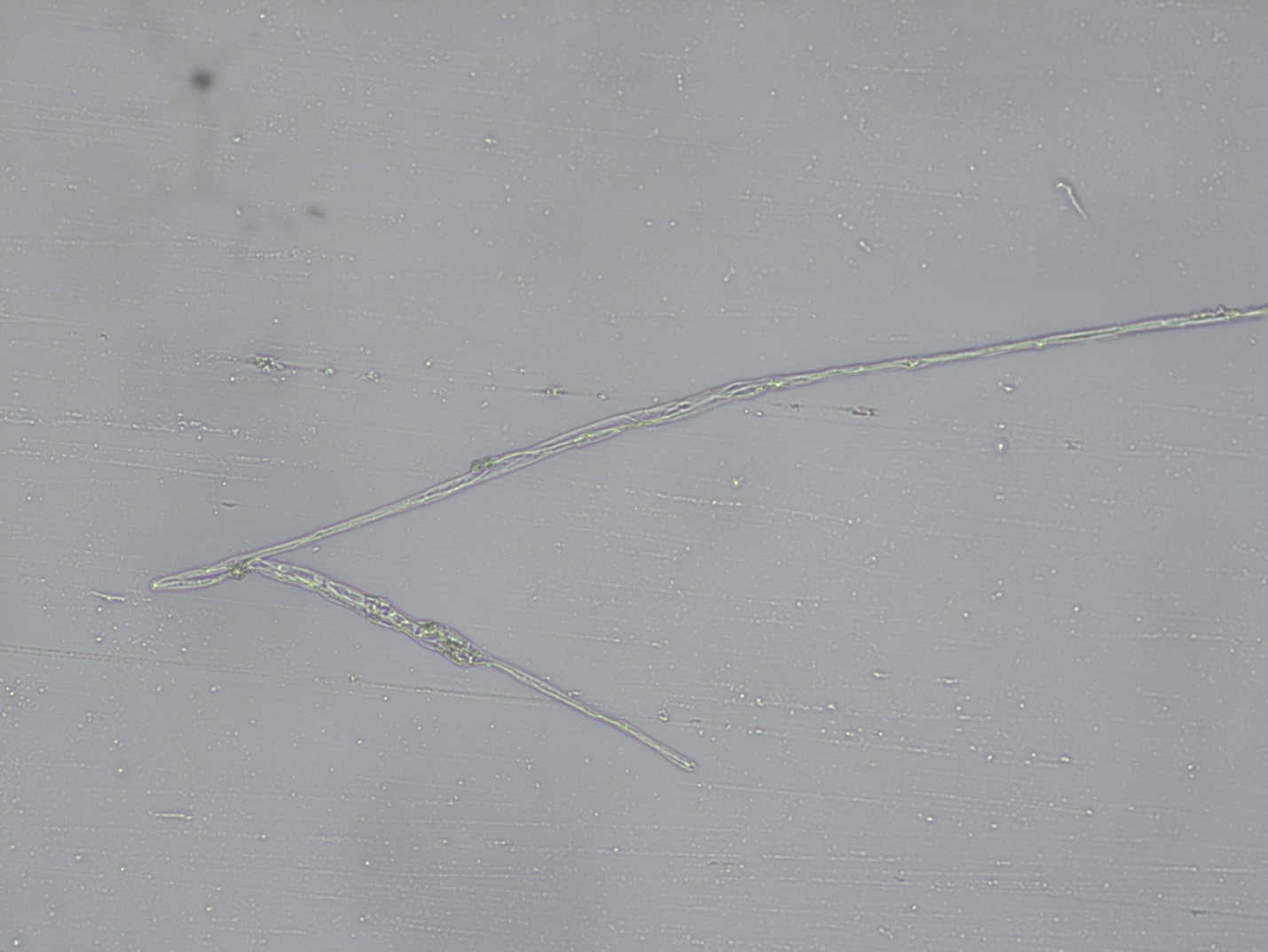


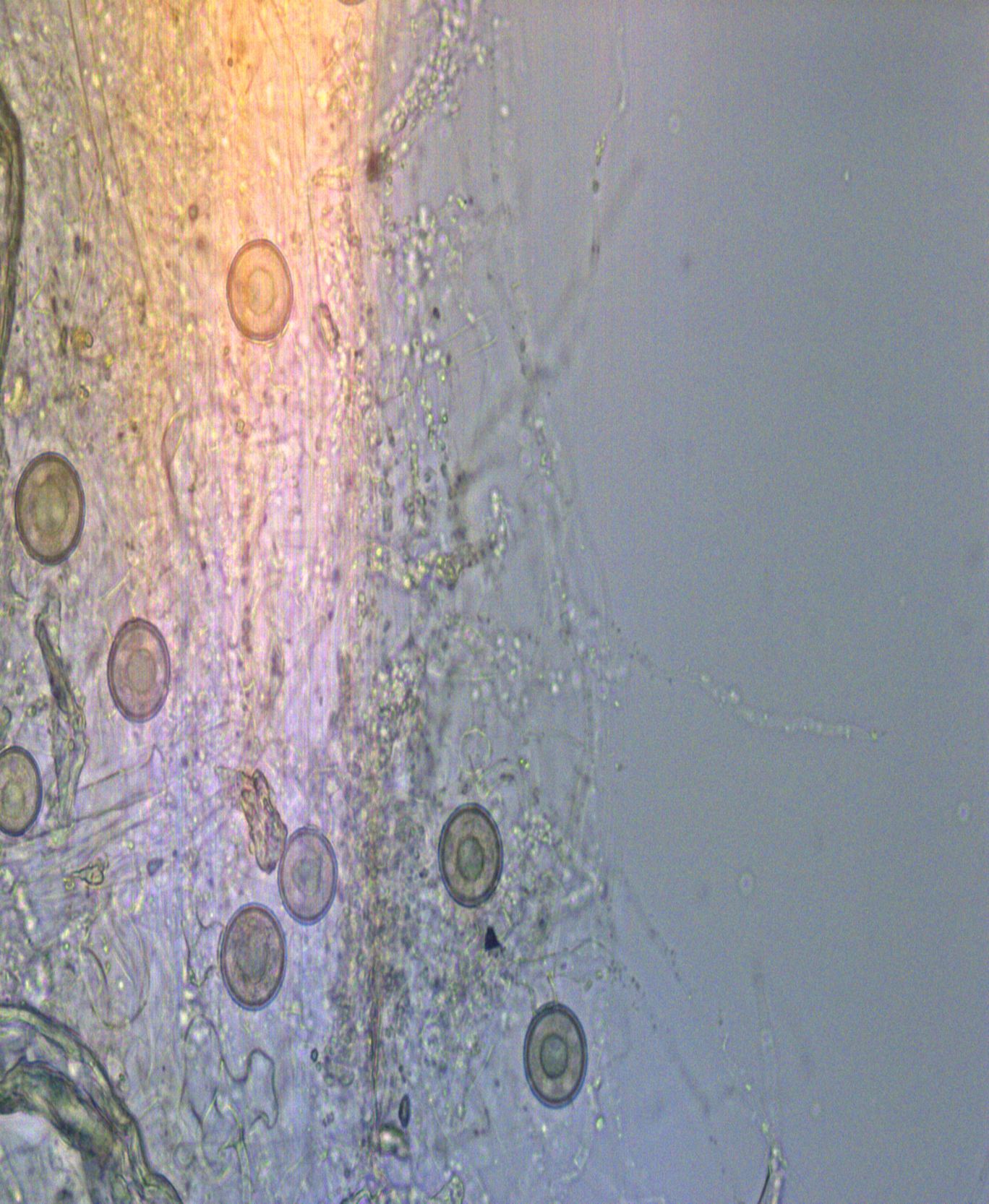

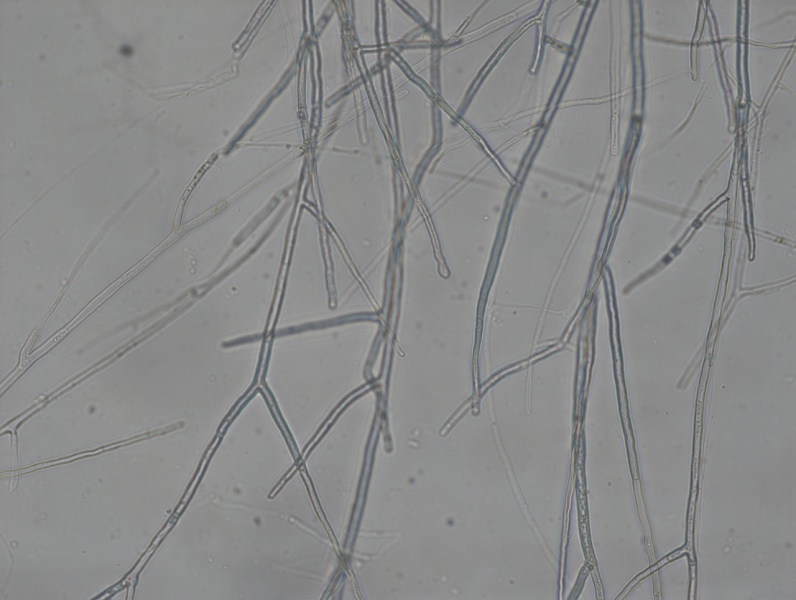


**c**

**b**

**a**

**e**

**d**

**Fig. S1.** Image illustrating the changes in morphological characters of *Pythium aphanidermatum* due to *in vitro* exposure of plant and microbial volatiles. a- *M. spicata* exposed culture, b- *C. citratus* exposed culture, c- *T. asperellum* exposed culture, d- control (Pathogenic culture) and e- sporulation of *P. aphanidermatum.*


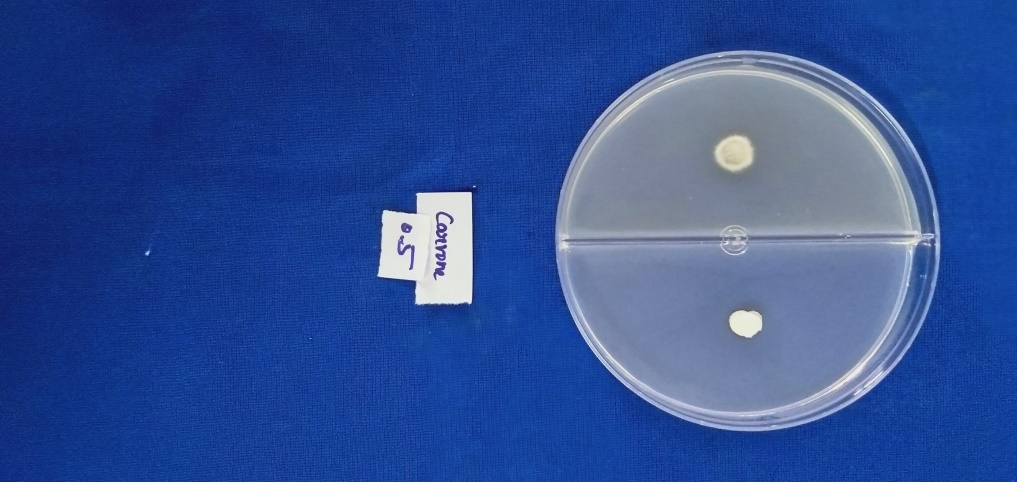

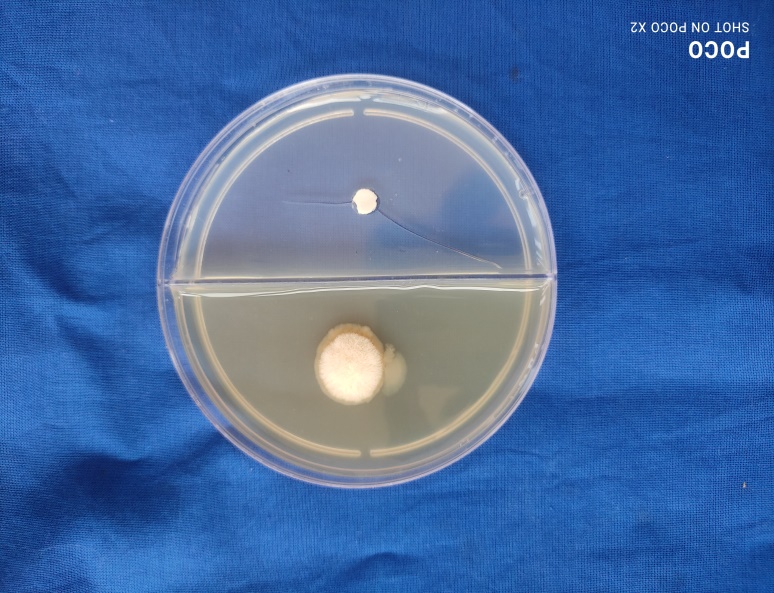

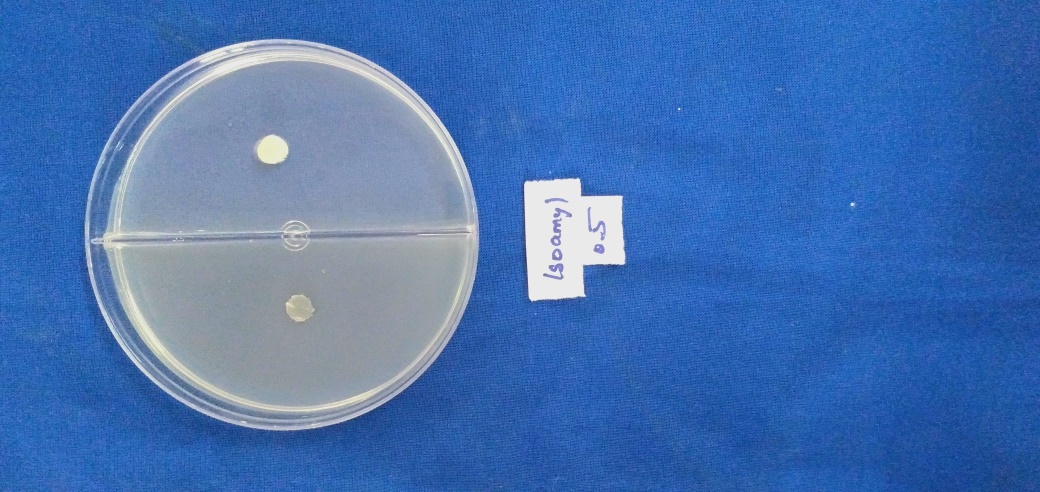


**c**

**b**

**a**


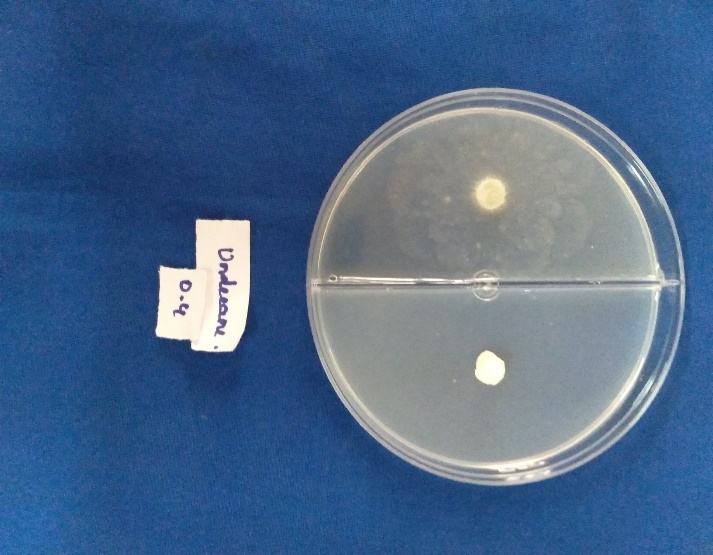

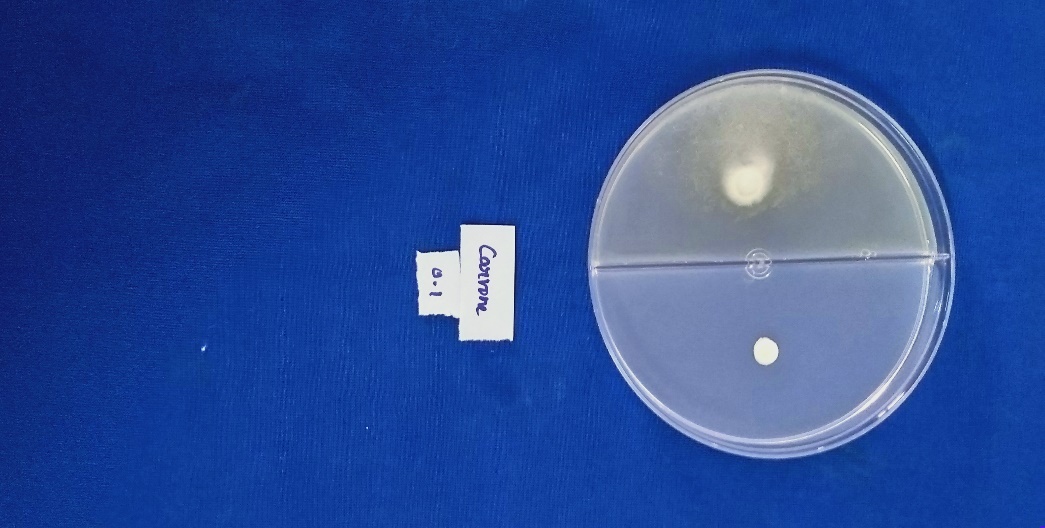

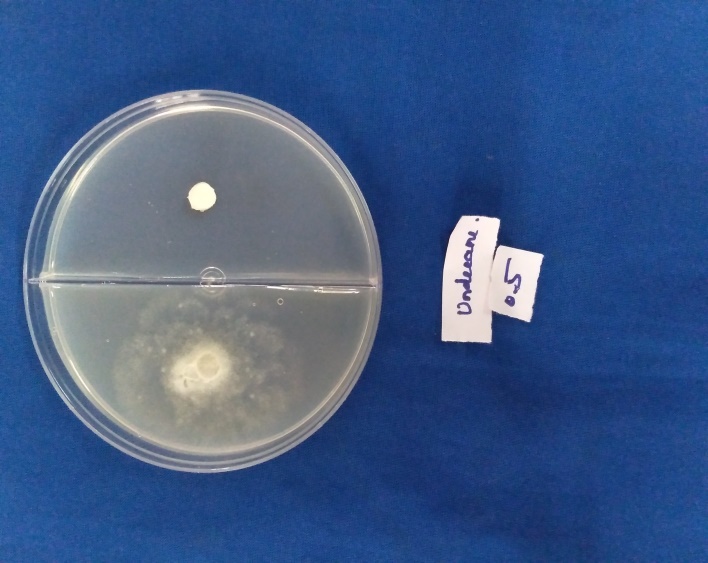


**f**

**e**

**d**

**
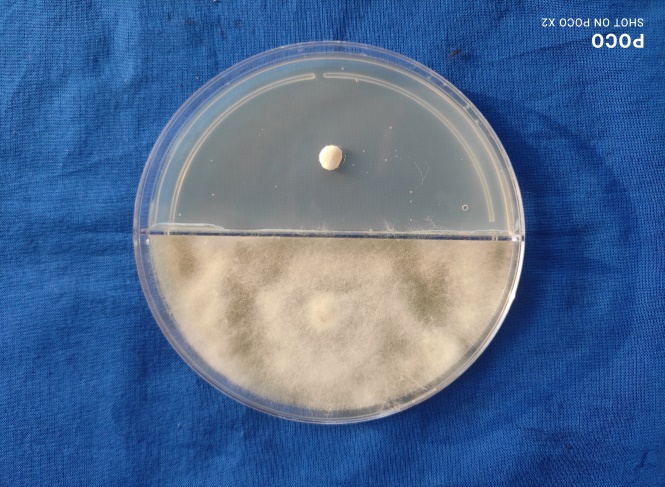

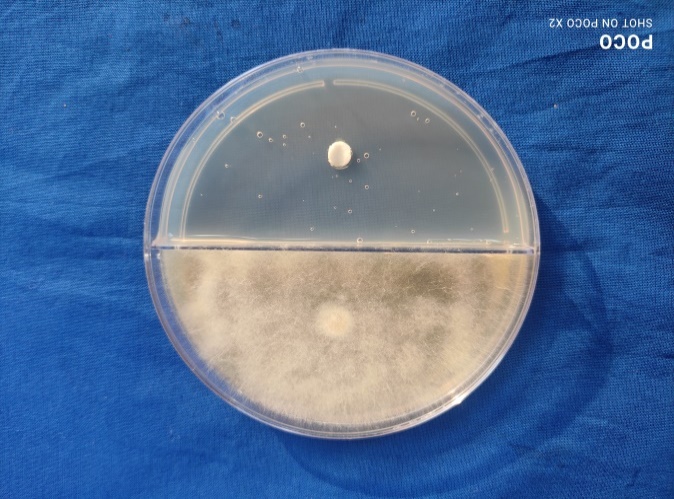
**

**h**

**g**

**Fig. S2.** Volatile action of hexane extract of *T. asperellum* (a)*, M. spicata* (b)*, C. citratus* (c)*,* VOCs of isopentyl alcohol (d), carvone (e) and citronellol (f) on *P. aphanidermatum* in bipartition plate. The image (g) represent hexane control and (h) pathogen control.

**Table S1.** Changes in the morphological characters of *Pythium aphanidermatum* due to *in vitro* exposure of plant volatilomes

| **Plant sample** | **Morphological characters** | | | | | | | | |
| --- | --- | --- | --- | --- | --- | --- | --- | --- | --- |
|  | **Mycelium** | | | **Sporangium** | | | **Oogonium**  **(cfu)** | **Antheridia**  **(cfu)** | **Oospores**  **(cfu)** |
|  | **Characters** | **Colour** | **W (µm)** | **Type** | **L**  **(µm)** | **W**  **(µm)** |  |  |  |
| *C. amboinicus* | Thick cottony mat | Colourless | 9.5-10 | Lobed | 28 - 30 | 17 | 3 | 2 | 4 |
| *O. tuniflorum* | Slow growth | Colourless | 10-11 | Terminal, lobed | 30 - 34 | 22 | 4 | 2 | 7 |
| *M. spicata* | Thin with less radiated pattern | Dull white | 2-3 | Absent | - | - | 0 | 0 | 0 |
| *C. citratus* | Irregular pattern of growth | Dirty white | 1.9-2.6 | Absent | - | - | 0 | 0 | 0 |
| *V. negundo* | Thick mat with profuse growth | Colourless | 10-12 | Lobed with smooth | 32 - 34 | 19 | 5 | 2 | 8 |
| *A. indica* | Regular pattern of growth | Dirty white | 9.5-11 | Filamentous with lobed | 32 | 20 | 4 | 3 | 7 |
| *V. zizanioides* | Thick cottony growth | Colourless | 9.8 | Lobed | 29.5 | 18.55 | 4 | 1 | 5 |
| Control | Thick cottony, profuse growth | Colourless | 12 – 12.5 | Branched, often lobed | 34 -36 | 26 | 8 | 4 | 7 |

L – Length, W - Width

**Table S2.** Changes in the morphological characters of *Pythium aphanidermatum* due to *in vitro* exposure of microbial volatilomes

| **Microbes** | **Morphological characters** | | | | | | | | |
| --- | --- | --- | --- | --- | --- | --- | --- | --- | --- |
|  | **Mycelium** | | | **Sporangium** | | | **Oogonium**  **(cfu)** | **Antheridia**  **(cfu)** | **Oospores**  **(cfu)** |
|  | **Characters** | **Colour** | **W**  **(µm)** | **Type** | **L**  **(µm)** | **W**  **(µm)** |  |  |  |
| *A. auriculata* | Thick cottony mat over grown | Dull white | 12.5 | Lobed, smooth | 29 | 16 | 6 | 3 | 5 |
| *C. cinereus* | Profuse cottony growth | Colourless | 11-12 | Small lobed | 21 | 13 | 7 | 2 | 6 |
| *G. lucidum* | Cottony mat over grown | Dull white | 12 | Sparse, rarely lobed | 23 - 24 | 15 | 5 | 3 | 7 |
| *L. edodes* | Cottony mycelial mat normal radiated pattern | Dirty white | 11-11.65 | Lobed | 34 | 23 | 4 | 2 | 5 |
| *T. asperellum* | Undulated poor mycelial mat with hyphal swellings | Dull white | 2.3 -2.8 | Absent | - | - | 0 | 0 | 0 |
| *B. subtilis* | Profuse mycelial mat, hyphal swellings | Colourless | 11.63 | Sparse with small lobes | 21-25 | 15.5 | 5 | 3 | 5 |
| *S. rochei* | Delicate with fast radiating pattern | Colourless | 12.2 | Inflated lobed, rough walled | 30.5 | 23.55 | 6 | 4 | 6 |
| Control | Thick cottony, profuse growth | Colourless | 12 – 12.5 | Branched, often lobed | 34 -36 | 26 | 8 | 4 | 7 |

L – Length, W - Width

| **RT** | **Compound** | **Molecular formula** | **Molecular weight** | **Relative abundance** |
| --- | --- | --- | --- | --- |
| 3.73 | 2-Penten-1-ol, 2-methyl- | C_6_H_12_O | 100 | 0.10 |
| 4.73 | 2,2,4-Trimethyl-3-pentanol | C_8_H_17_N | 127 | 0.78 |
| 5.52 | Pentanoic acid, 2,2,4-trimethyl-3-hydroxy-, isobutyl ester | C_16_H_30_O_4_ | 286 | 0.25 |
| 6.79 | Cyclohexanol, 2-methyl-5-(1-methylethenyl)-, (1à,2à,5á)- | C_7_H_14_O | 114 | 0.20 |
| 8.27 | 10,13-Octadecadiynoic acid, methyl ester | C_19_H_30_O_2_ | 219 | 0.00 |
| 9.86 | trans-Carveol | C_10_H_16_O | 152 | 0.12 |
| 10.25 | (-)-Carvone | C_10_H_14_O | 150 | 3.08 |
| 11.55 | 10,13-Octadecadiynoic acid, methyl ester | C_19_H_30_O_2_ | 219 | 0.02 |
| 11.55 | Limonen-6-ol, pivalate | C_20_H_32_O_3_ | 320 | 0.02 |
| 11.92 | Ethyl iso-allocholate | C_26_H_44_O_5_ | 436 | 0.01 |
| 12.37 | (-)-á-Bourbonene | C_15_H_24_ | 204 | 1.67 |
| 12.9 | Caryophyllene | C_15_H_24_ | 204.18 | 1.57 |
| 13.31 | alfa.-Copaene | C_15_H_24_ | 204 | 0.25 |
| 13.63 | (+)-epi-Bicyclosesquiphellandrene | C_15_H_24_ | 204 | 0.84 |
| 13.96 | 1,6-Cyclodecadiene,1-methyl-5-methylene-8-(1-methylethyl)-, [S-(E,E)]- | C_15_H_24_ | 204 | 1.57 |
| 14.67 | Naphthalene,1,2,3,4-tetrahydro-1,6-dimethyl-4-(1-methylethyl)-, (1S-cis)- | C_10_H_4_C_l4_ | 264 | 0.51 |
| 15.24 | Rhodopin | C_40_H_58_O | 554 | 0.01 |
| 15.71 | Tetradecane, 2,6,10-trimethyl- | C_17_H_36_ | 240 | 0.13 |
| 16.18 | Ethyl iso-allocholate | C_26_H_44_O_5_ | 436 | 0.01 |
| 17.46 | Benzoic acid, 2-ethylhexyl ester | C_15_H_22_O_2_ | 234 | 0.09 |
| 18.54 | Octadecane | C_18_H_38_ | 254 | 0.05 |
| 18.89 | Cyclononasiloxane, octadecamethyl- | C_18_H_54_O_9_Si_9_ | 666 | 0.06 |
| 19.46 | Phthalic acid, hept-4-yl isobutyl ester | C_31_H_52_O_4_ | 488 | 0.10 |
| 20.6 | 9,12,15-Octadecatrienoic acid, | C_18_H_32_O | 264 | 0.12 |
| 21.5 | 9-Desoxo-9-x-acetoxy-3,8,12-tri-O-acetylingol | C_28_H_40_O_10_ | 536 | 0.03 |

**Table S3.** GC-MS profiling of VOCs produced by leaves of *Mentha spicata*

| **RT** | **Compound** | **Molecular formula** | **Molecular weight** | **Relative area abundance** |
| --- | --- | --- | --- | --- |
| 2.89 | Oxirane, 2-ethyl-2-methyl- | C_5_H_10_O | 86 | 0.87 |
| 4.75 | 2,2,4-Trimethyl-3-pentanol | C_8_H_17_N | 127 | 0.38 |
| 5.12 | Hydroperoxide, 1-ethylbutyl | C_6_H_14_O_2_ | 118 | 0.34 |
| 5.54 | Oxirane, butyl- | C_6_H_12_O | 100 | 0.72 |
| 7.03 | 3-Carene | C_10_H_16_ | 136 | 0.05 |
| 7.95 | 1,6-Octadien-3-ol, 3,7-dimethyl- | C_10_H_18_O | 154 | 0.07 |
| 8.78 | Isopulegol | C_10_H_18_O | 154 | 0.16 |
| 9.97 | Citronellol | C_10_H_20_O | 156 | 5.27 |
| 10.37 | Geraniol | C_10_H_18_O | 154 | 15.85 |
| 11.02 | Geranyl vinyl ether | C_12_H_20_O | 180 | 0.00 |
| 12.13 | 2,6-Octadien-1-ol, 3,7-dimethyl-, acetate | C_10_H_18_O | 154 | 1.20 |
| 12.9 | Caryophyllene | C_15_H_24_ | 204 | 0.13 |
| 14.55 | ç-Muurolene | C_15_H_24_ | 204 | 2.09 |
| 15.32 | à-acorenol | C_15_H_26_O | 222 | 0.00 |
| 15.59 | 4-epi-cubedol | C_22_H_32_O_2_ | 328 | 0.63 |
| 16.22 | Cubedol | C_23_H_22_O_6_ | 394 | 0.03 |
| 16.79 | à-Cadinol | C_15_H_26_O | 222 | 0.13 |
| 17.46 | Benzoic acid, 2-ethylhexyl ester | C_15_H_22_O_2_ | 234 | 0.18 |
| 17.97 | Geranyl isovalerate | C_15_H_26_O_2_ | 238 | 0.04 |
| 18.54 | Octadecane | C_18_H_38_ | 254 | 0.04 |
| 18.54 | Heptacosane | C_20_H_60_O_10_Si_10_ | 740 | 0.04 |
| 19.05 | Phytol, acetate | C_22_H_42_O_2_ | 338 | 0.24 |
| 19.46 | Dibutyl phthalate | C_16_H_22_O_4_ | 278 | 0.16 |
| 19.93 | Betulin | C_30_H_50_O_2_ | 442 | 0.03 |
| 21.48 | Ethyl iso-allocholate | C_26_H_44_O_5_ | 436 | 0.01 |
| 22.62 | Cyclodecasiloxane, eicosamethyl- | C_20_H_60_O_10_Si_10_ | 740 | 0.03 |

**Table S4.** GC-MS profiling of VOCs produced by leaves of *Cymbopogon citratus*

**Table S5.** GC-MS profiling and volatile composition of *T. asperellum*

| **RT** | **Compound** | **Molecular weight** | **Relative area abundance** |
| --- | --- | --- | --- |
| 10.04 | 5,8,11-Heptadecatriynoic acid, methyl ester | 272 | 0.70 |
| 10.10 | Isopentyl alcohol | 88 | 3.40 |
| 12.27 | dl-Limonene | 136 | 1.20 |
| 13.43 | 2H-Pyran-2-one, 6-pentyl- | 166 | 0.45 |
| 14.02 | Tetradecane, 2,6,10-trimethyl- | 240 | 0.31 |
| 15.08 | cis-9,10-Epoxyoctadecanamide | 284 | 0.22 |
| 15.73 | Tetradecane, 2,6,10-trimethyl- | 240 | 0.23 |
| 16.34 | 7-epi-cis-sesquisabinene hydrate | 222 | 0.25 |
| 16.73 | Ethyl iso-allocholate | 436 | 0.11 |
| 17.26 | 4a,10a-Methanophenanthren-9á-ol, | 290 | 0.16 |
| 18.14 | 1,3,6,10-Cyclotetradecatetraene, | 272 | 0.39 |
| 18.54 | Tetradecane, 2,6,10-trimethyl- | 240 | 0.45 |
| 19.46 | 1,2-Benzenedicarboxylic acid, bis(2-methylpropyl) ester | 166 | 0.45 |
| 20.01 | Phthalic acid, butyl hept-4-yl ester | 320 | 1.11 |
| 20.52 | Dibutyl phthalate | 278 | 0.97 |
| 21.01 | 1,3,6,10-Cyclotetradecatetraene, | 272 | 0.42 |
| 21.44 | Bicyclo[9.3.1]pentadeca-3,7-dien-12-ol, | 290 | 0.07 |
| 21.90 | 2-[4-methyl-6-(2,6,6-trimethylcyclohex-1-enyl)hexa-1,3,5-trimethyl | 324 | 0.09 |
| 22.37 | Cyclodecasiloxane, eicosamethyl- | 740 | 0.09 |
| 22.92 | Cyclodecasiloxane | 740 | 0.01 |
